# Limiting weight gain in overweight and obese women during pregnancy to improve health outcomes: the LIMIT randomised controlled trial

**DOI:** 10.1186/1471-2393-11-79

**Published:** 2011-10-26

**Authors:** Jodie M Dodd, Deborah A Turnbull, Andrew J McPhee, Gary Wittert, Caroline A Crowther, Jeffrey S Robinson

**Affiliations:** 1 Discipline of Obstetrics and Gynaecology, School of Paediatrics and Reproductive Health, The University of Adelaide, Adelaide, Australia; 2 School of Psychology, The University of Adelaide, Adelaide, Australia; 3 Department of Perinatal Medicine, The Women's and Children's Hospital, Adelaide, Australia; 4 School of Medicine, The University of Adelaide, Adelaide, Australia

## Abstract

**Background:**

Obesity is a significant global health problem, with the proportion of women entering pregnancy with a body mass index greater than or equal to 25 kg/m^2 ^approaching 50%. Obesity during pregnancy is associated with a well-recognised increased risk of adverse health outcomes both for the woman and her infant, however there is more limited information available regarding effective interventions to improve health outcomes.

The aims of this randomised controlled trial are to assess whether the implementation of a package of dietary and lifestyle advice to overweight and obese women during pregnancy to limit gestational weight gain is effective in improving maternal, fetal and infant health outcomes.

**Methods/Design:**

Design: Multicentred randomised, controlled trial.

Inclusion Criteria: Women with a singleton, live gestation between 10^+0^-20^+0 ^weeks who are obese or overweight (defined as body mass index greater than or equal to 25 kg/m^2^), at the first antenatal visit.

Trial Entry & Randomisation: Eligible, consenting women will be randomised between 10^+0 ^and 20^+0 ^weeks gestation using a central telephone randomisation service, and randomisation schedule prepared by non-clinical research staff with balanced variable blocks. Stratification will be according to maternal BMI at trial entry, parity, and centre where planned to give birth.

Treatment Schedules: Women randomised to the *Dietary and Lifestyle Advice Group *will receive a series of inputs from research assistants and research dietician to limit gestational weight gain, and will include a combination of dietary, exercise and behavioural strategies.

Women randomised to the *Standard Care Group *will continue to receive their pregnancy care according to local hospital guidelines, which does not currently include routine provision of dietary, lifestyle and behavioural advice.

Outcome assessors will be blinded to the allocated treatment group.

Primary Study Outcome: infant large for gestational age (defined as infant birth weight ≥ 90^th ^centile for gestational age).

Sample Size: 2,180 women to detect a 30% reduction in large for gestational age infants from 14.40% (p = 0.05, 80% power, two-tailed).

**Discussion:**

This is a protocol for a randomised trial. The findings will contribute to the development of evidence based clinical practice guidelines.

**Trial Registration:**

Australian and New Zealand Clinical Trials Registry ACTRN12607000161426

## Background

### Obesity: the burden of disease

Obesity has been identified by the World Health Organisation as a significant global health problem,[1] contributing to the overall burden of disease worldwide [2]. Obesity is associated with a reduction in life expectancy, through an increased risk of adverse health consequences related to cardiovascular disease, type 2 diabetes and several cancers [2].

### The impact of obesity during pregnancy

Obesity is a significant health issue for women during pregnancy and childbirth, with estimates suggesting that 35% of Australian women aged between 25 and 35 years are overweight or obese [3]. Approximately 34% of pregnant women have a body mass index (BMI) in excess of 25 kg/m^2^,[4] although recent data indicates this to be increasing further, and approaching 50% [5,6]. There are well-documented risks associated with obesity during pregnancy. Maternal complications include an increased risk of hypertension and pre-eclampsia,[4-17] gestational diabetes,[4-6,10,11,15,16] infection[13] and thromboembolic disease [9,13]. Women who are overweight or obese are more likely to require induction of labour,[5,6,10,13,14,16] and have increased risk of caesarean section [4-6,10-18]. Women who are overweight or obese are more likely to suffer a perinatal death [4,10,12,14,17-22]. Infants of mothers who are overweight or obese are more likely to be macrosomic,[5,6,15,17,19-21] require admission to the neonatal intensive care unit,[4,6,19-21] be born preterm,[4,6,19-21] be identified with a congenital anomaly,[4,19-21] and to require treatment for jaundice or hypoglycaemia [4,6,16].

We have published maternal and infant health outcome data available for all women birthing in South Australia in 2008, involving in excess of 19,600 confinements [6]. Risk ratios (RR) and 95% confidence intervals (CI) for health outcomes for women in various BMI categories were compared with women of healthy BMI (less than 25 kg/m^2^), demonstrating an increased risk in adverse maternal and infant health outcomes with increasing maternal BMI,[6] consistent with other reported literature [4,5].

### Gestational weight gain recommendations

There is a substantial literature on maternal weight gain in pregnancy that has been summarised by the Institute of Medicine [23,24]. While most studies have reported average weight gains of 10-15 kg, this varied considerably among overweight and obese women, with many women exceeding this average [23].

Cedergren and colleagues evaluated the effect of maternal BMI and gestational weight gain in more than 240,000 women in Sweden [25]. While obese women with gestational weight gain less than 8 kg had a reduced risk of large-for-gestational age babies, pre-eclampsia, caesarean section and operative vaginal birth, high gestational weight gain was associated with an increased rate of caesarean section across each of the 5 BMI categories. Cedergren concluded that obese women may benefit from a limited weight gain in pregnancy, as has been recommended by the Institute of Medicine [23,24].

Conversely, too severe restriction of maternal diet may have implications for fetal growth and development. While Cedergren and colleagues demonstrated a reduction in the risk of large for gestational age infants among women with less than 8 kg weight gain in pregnancy, this appeared to be at the expense of an increase in the proportion of small infants at the opposite end of the birth-weight spectrum [25]. This finding has not been demonstrated in the randomised trial ACHOIS[13] where, for women with gestational diabetes, treatment with dietary and exercise advice designed not to promote weight loss was associated with a significant reduction in the incidence of macrosomia without any increase in the risk of small for gestational age infants.

While restriction of weight gain in pregnancy increases the incidence of spontaneous preterm birth in women with a normal BMI, this has not been demonstrated in overweight or obese women [26]. Thus recommendations have been made to avoid weight loss during pregnancy and to limit restriction of weight gain to approximately 5 kg throughout pregnancy [27].

### Reducing the burden of disease related to Obesity in Pregnancy

While there is an extensive body of literature related to defining the problems and potential complications associated with obesity during pregnancy and childbirth, there is more limited information available related to effective interventions that may be implemented to improve maternal, fetal and infant health outcomes. Current practice guidelines recommend that women should be counselled prior to conception and encouraged to make lifestyle changes [28].

### Systematic Review of the Literature

Our systematic review published in 2008[29] identified 2 published randomised trials, assessing antenatal interventions for overweight and obese women during pregnancy. Our updated systematic review and meta-analysis published in 2010 included 9 randomised trials, involving 743 women and infants [30]. There were no statistically significant differences identified between women who received an antenatal intervention and those who did not for the outcome large-for-gestational-age infant (three studies; 366 women; risk ratio 2.02; 95% CI 0.84, 4.86) or mean gestational weight gain (four studies; 416 women; weighted mean difference -3.10 kg; 95% CI -8.32, 2.13 (random effects model). There were no statistically significant differences identified for other reported outcomes.

### To summarise

There are limitations of the current evidence on the effect of an antenatal dietary intervention to limit gestational weight gain for women who are overweight or obese, including the lack of reporting of maternal and infant outcomes.

#### Aims of the trial

The aims of this randomised, controlled trial are to assess whether the implementation of a package of dietary and lifestyle advice to overweight and obese women during pregnancy to limit weight gain is effective in improving maternal, fetal and infant health outcomes.

#### Trial hypotheses

**The primary hypothesis **is that the implementation of a package of dietary and lifestyle advice to overweight and obese women during pregnancy to limit gestational weight gain will

• **reduce the risk of the infant born large for gestational age **(defined as infant birth weight ≥ 90^th ^centile for gestational age and infant sex).

**The secondary hypotheses **are that the implementation of a package of dietary and lifestyle advice to overweight and obese women during pregnancy to limit gestational weight gain will

• **reduce the risk of morbidity from other adverse outcomes for the infant; and**

• **reduce the risk of maternal morbidity from pregnancy complications and adverse outcomes**.

## Methods/Design

Study Design: Multicentred randomised controlled trial.

Inclusion Criteria: Women with a singleton, live gestation between 10^+0^-20^+0 ^weeks who are obese or overweight (defined as a body mass index greater than or equal to 25 kg/m^2^), at their first antenatal visit.

Exclusion Criteria: Women with a multiple pregnancy, or type 1 or 2 diabetes diagnosed prior to pregnancy.

Trial Entry: Eligible women will be identified in the antenatal clinic, given the trial information sheet and counselled by the researcher, before obtaining informed written consent. Randomisation will occur between 10^+0 ^and 20^+0 ^weeks gestation by telephoning the central randomisation service. The randomisation schedule will use balanced variable blocks, and will be prepared by an investigator not involved with recruitment or clinical care. There will be stratification of women according to parity (parity 0 versus parity 1 or more), body mass index at booking visit (BMI 25-29.9 kg/m^2 ^versus BMI ≥ 30 kg/m^2^), and collaborating centre.

Treatment Schedules: Eligible women will be randomised and allocated a study number corresponding to either the '**Dietary and Lifestyle Advice Group**' or the '**Standard Care Group**'.

Women randomised to the **Dietary and Lifestyle Advice Group **will receive a comprehensive intervention to limit weight gain in pregnancy that includes a combination of dietary, exercise and behavioural strategies, delivered by a research dietician and trained research assistants. Women will be provided with dietary advice consistent with current Australian dietary standards,[31] that maintains a balance of carbohydrates, fat and protein. Women will be encouraged to reduce their intake of foods high in refined carbohydrates and saturated fats, while increasing their intake of fibre, and promoting consumption of two serves of fruit and five serves of vegetables each day [31]. Exercise advice will focus on encouraging women to adopt a more active lifestyle, primarily by increasing the amount of walking [32]. Tailoring of the intervention will be informed by stage theories of health decision making that propose that individuals progress through a series of cognitive phases when undertaking behavioural change [33].

Initially, there will be a planning session with a research dietician, in which women are provided with written dietary and activity information, an individual diet and physical activity plan, a food and activity diary, recipe book and example menu plans. Women will be encouraged to set achievable goals for dietary and exercise change, supported to make these lifestyle changes and to self-monitor their progress. Additionally, women will be encouraged to identify potential barriers and enablers to facilitate the implementation of their goals. This information will be reinforced during subsequent inputs at regular intervals during pregnancy, provided by the research dietician (at 28 weeks' gestation) and trained research assistants (via telephone call at 22, 24, and 32 weeks' gestation and a face-face visit at 36 weeks' gestation).

Women randomised to the *Standard Care Group *will continue to receive their pregnancy care according to local hospital guidelines, which does not currently include routine provision of dietary, lifestyle and behavioural advice.

Follow-up of women in both treatment groups: All women will continue to have their clinical care provided by clinicians at their planned hospital of birth. After birth, information will be obtained relating to birth and infant outcomes from the case notes by a research assistant not involved in the provision of the intervention, and blinded to treatment allocation. Similarly, the postnatal and neonatal forms will be completed for each live born infant after discharge from hospital. Outcomes will be determined at the time of primary hospital discharge or at 6 weeks post-partum, whichever is greater.

Study Endpoints: The primary study outcome is:

• **Infant large for gestational age **(defined as birth weight ≥ 90^th ^centile for gestational age).

The secondary study outcomes are

**1) Adverse outcomes for the infant **including preterm birth (defined as birth at less than 37 weeks gestation); mortality (defined as either a stillbirth (intrauterine fetal death after trial entry and prior to birth), or infant death (death of a live born infant prior to hospital discharge, and excluding lethal congenital anomalies)); congenital anomalies; infant birth weight ≥ 4000 grams; hypoglycaemia requiring intravenous treatment; admission to neonatal intensive care unit, or special care baby unit; hyperbilirubinaemia requiring phototherapy; nerve palsy; fracture; birth trauma; shoulder dystocia.

**2) Adverse outcomes for the woman **including maternal hypertension and pre-eclampsia (in accordance with recognised Australasian Society for the Study of Hypertension in Pregnancy criteria)[34]; maternal gestational diabetes (defined as a positive oral glucose tolerance test with fasting blood glucose level ≥ 5.5 mmol/L, or 2 hour blood glucose level ≥ 7.8 mmol/L); need for and length of antenatal hospital stay; antepartum haemorrhage requiring hospitalisation; preterm prelabour ruptured membranes; chorioamnionitis requiring antibiotic use during labour; need and reason for induction of labour; any antibiotic use during labour; caesarean section; postpartum haemorrhage (defined as blood loss ≥ 600 mL); perineal trauma; wound infection; endometritis; use of postnatal antibiotics; length of postnatal hospital stay; thromboembolic disease; maternal death.

Sample Size: The clinical endpoint of infant large for gestational age has been chosen as the primary outcome for this trial. Population studies consistently identify high infant birth weight as a significant, independent factor associated with a 2-3 fold increased odds of obesity at age 4 yrs,[35,36] and more accurately assesses size at birth than weight above a particular cut-off value. We estimate the incidence of infant large for gestational age in women eligible for this trial to be 14.4% [37]. To reduce this by 30% to 10.1% (alpha 0.05; power 80%; two-tailed test), and allowing for 15% loss to follow-up, we will need to recruit a total of 2,180 women.

Analysis and Reporting of Results: The initial analysis will examine baseline characteristics of all randomised women, as an indication of comparable treatment groups. Outcome comparisons for women and infants will be analysed for the primary and secondary outcomes on an "intention to treat" basis, according to treatment allocation at randomisation to either dietary and lifestyle group or standard care group. The relative risks and 95% confidence intervals will be reported for the major outcomes, and the number needed to treat to prevent one adverse outcome will be calculated. Regression techniques will be used to examine the influence of prognostic factors on the major outcomes. Pre-specified secondary analyses will explore the effect of maternal BMI, gestational weight gain and parity on clinical outcomes.

Approval to conduct this study has been obtained from the following research and ethics committees: Women's and Children's Hospital (Adelaide, South Australia); Flinders Medical Centre (Adelaide, South Australia); Lyell McEwin Health Service (Adelaide, South Australia); The Queen Elizabeth Hospital (Adelaide, South Australia).

## Discussion

This is a protocol for a randomised trial assessing whether the implementation of a package of dietary and lifestyle advice to overweight and obese women during pregnancy to limit gestational weight gain is effective in improving maternal, fetal and infant health outcomes. The findings of this trial will contribute to the currently available literature regarding the effect of limiting gestational weight gain for women who are overweight or obese, and to the development of evidence based clinical practice guidelines.

## Competing interests

The authors declare that they have no competing interests.

## Authors' contributions

JMD, DT, AJM, GW, CAC, JSR all contributed to the development of the trial protocol. JMD drafted this manuscript and all authors reviewed critically for content and gave approval to the final to be published version of the manuscript.

## Pre-publication history

The pre-publication history for this paper can be accessed here:

http://www.biomedcentral.com/1471-2393/11/79/prepub

## References

[B1] World Health Organisation WHO Obesity: preventing and managing the global epidemic. WHO Technical Report Series Number 894 2000 Geneva: World Health Organisation 11234459

[B2] Ezzati M Lopez AD Rodgers A Vander Hoorn S Murray CJ the Comparative Risk Assessment Collaborating Group Selected major risk factors and global and regional burden of disease Lancet 2002 360 1347 1360 10.1016/S0140-6736(02)11403-6 12423980

[B3] Cameron AJ Welborn TA Zimmet PZ Overweight and obesity in Australia: the 1999-2000 Australian Diabetes, Obesity and Lifestyle Study MJA 2003 178 427 432 12720507 10.5694/j.1326-5377.2004.tb05998.x

[B4] Callaway LK Prins JB Chang AM McIntyre HD The prevalence and impact of overweight and obesity in an Australian obstetric population MJA 2006 184 2 56 59 16411868 10.5694/j.1326-5377.2006.tb00115.x

[B5] Athukorala C Rumbold AR Willson KJ Crowther CA The risk of adverse pregnancy outcomes in women who are overweight or obese BMC Pregnancy and Childbirth 2010 10 56 10.1186/1471-2393-10-56 20849609 PMC2949787

[B6] Dodd JM Grivell RM Nguyen A-M Chan A Robinson JS Maternal and perinatal health outcomes by body mass index category ANZJOG 2011 51 2 136 140 21466515 10.1111/j.1479-828X.2010.01272.x

[B7] Sibai BM Gordon T Thorn E Risk factors for preeclampsia in healthy nulliparous women: a prospective multicentre study American Journal of Obstetrics and Gynecology 1995 172 2 part 1 642 648 7856699 10.1016/0002-9378(95)90586-3

[B8] Ness RB Roberts JM Heterogeneous causes constituting the single syndrome of preeclampsia: a hypothesis and its implications American Journal of Obstetrics and Gynecology 1996 175 1365 1370 10.1016/S0002-9378(96)70056-X 8942516

[B9] Wolfe H High prepregnancy body-mass index - a maternal-fetal risk factor New England Journal of Medicine 1998 338 191 192 10.1056/NEJM199801153380310 9428822

[B10] Sebire NJ Harris JP Wadsworth J Joffe M Beard RW Regan L Robinson S Maternal obesity and pregnancy outcome: a study of 287,213 pregnancies in London Int J Obes Relat Metab Disord 2001 25 8 1175 1182 10.1038/sj.ijo.0801670 11477502

[B11] La Coursiere DY Bloebaum L Duncan JD Varner MW Population-based trends and correlates of maternal overweight and obesity, Utah 1991-2001 American Journal of Obstetrics and Gynecology 2005 192 832 839 10.1016/j.ajog.2004.11.034 15746679

[B12] Nohr EA Bech BH Davies MJ Frydenberg M Henriksen TB Olsen J Prepregnancy obesity and fetal death: a study within the Danish National Birth Cohort Obstet Gynecol 2005 106 2 250 259 10.1097/01.AOG.0000172422.81496.57 16055572

[B13] Crowther CA Hiller JE Moss JR McPhee AJ Jeffries WS Robinson JS Group ftACISiPWAT Effect of treatment of gestational diabetes mellitus on pregnancy outcomes New England Journal of Medicine 2005 352 24 2477 2486 10.1056/NEJMoa042973 15951574

[B14] Usha Kiran TS Hemmadi S Bethel J Evans J Outcome of pregnancy in a woman with an increased body mass index British Journal of Obstetrics and Gynaecology 2005 112 768 772 10.1111/j.1471-0528.2004.00546.x 15924535

[B15] Abenhaim HA Kinch RA Morin L Benjamin A Usher R Effect of prepregnancy body mass index categories on obstetrical and neonatal outcomes Arch Gynecol Obstet 2007 275 39 43 16967276 10.1007/s00404-006-0219-y

[B16] Doherty DA Magann EF Francis J Morrison JC Newnham JP Pre-pregnancy body mass index and pregnancy outcomes International Journal of Gynecology and Obstetrics 2006 95 242 247 10.1016/j.ijgo.2006.06.021 17007857

[B17] Cedergren MI Maternal morbid obesity and the risk of adverse pregnancy outcome Obstet Gynecol 2004 103 219 224 10.1097/01.AOG.0000107291.46159.00 14754687

[B18] Kristensen J Vestergaard M Wisborg K Kesmodel U Secher NJ Pre-pregnancy weight and the risk of stillbirth and neonatal death British Journal of Obstetrics and Gynaecology 2005 112 403 408 10.1111/j.1471-0528.2005.00437.x 15777435

[B19] Cnattingius S Bergstrom R Lipworth L Kramer MS Prepregnancy weight and the risk of adverse pregnancy outcomes New England Journal of Medicine 1998 338 147 152 10.1056/NEJM199801153380302 9428815

[B20] Galtier-Dereure F Boegner C Bringer J Obesity and pregnancy: complications and cost Am J Clin Nutr 2000 71 S1242 S1248 10.1093/ajcn/71.5.1242s 10799397

[B21] Rosenberg TJ Garbers S Chavkin W Chiasson MA Prepregnancy weight and adverse perinatal outcomes in an ethnically diverse population Obstet Gynecol 2003 102 5 Part 1 1022 1027 14672480 10.1016/j.obstetgynecol.2003.07.005

[B22] Fretts RC Etiology and prevention of stillbirth American Journal of Obstetrics and Gynecology 2005 193 6 1923 1935 10.1016/j.ajog.2005.03.074 16325593

[B23] Institute of Medicine Nutrition during pregnancy 1990 Washington DC: National Academy Press

[B24] Institute of Medicine Rasmussen KM, Yaktine AL Weight gain during pregnancy: reexamining the guidelines 2009 Washington D.C. 20669500

[B25] Cedergren MI Effects of gestational weight gain and body mass index on obstetric outcomes in Sweden International Journal of Gynecology and Obstetrics 2006 93 269 274 10.1016/j.ijgo.2006.03.002 16626716

[B26] Schieve LA Cogswell ME Scanlon KS Perry G Ferre C Blackmore-Prince C Yu SM Rosenberg D Prepregnancy body mass index and pregnancy weight gain: associations with preterm delivery Obstet Gynecol 2000 96 194 200 10.1016/S0029-7844(00)00883-8 10908762

[B27] Rae A Bond D Evans SF North F Roberman B Walters B A randomised controlled trial of dietary energy restriction in the management of obese women with gestational diabetes ANZJOG 2000 40 4 416 422 11194427 10.1111/j.1479-828x.2000.tb01172.x

[B28] American College of Obstetricians and Gynecologists ACOG ACOG Committee Opinion number 315, September 2005: Obesity in pregnancy Obstet Gynecol 2005 106 3 671 675 10.1097/00006250-200509000-00054 16135613

[B29] Dodd JM Crowther CA Robinson JS Dietary and lifestyle interventions to limit weight gain during pregnancy for obese or overweight women: a systematic review Acta Obstet Gynecol Scand 2008 2 1 5 (Epub) 10.1080/00016340802061111 18607830

[B30] Dodd JM Grivell RM Crowther CA Robinson JS Antenatal interventions for overweight or obese pregnant women: a systematic review of randomised trials British Journal of Obstetrics and Gynaecology 2010 117 11 1316 1326 10.1111/j.1471-0528.2010.02540.x 20353459

[B31] The Australian Guide to Healthy Eating http://www.health.gov.au/internet/main/publishing.nsf/content/health-pubhlth-publicat-document-fdcons-cnt.htm

[B32] Royal College of Obstetricians and Gynaecologists RCOG Recreational exercise and pregnancy: information for you 2006 RCOG Press

[B33] Bennett P Murphy S Psychology and health promotion 1997 Buckingham: Open University Press

[B34] Brown MA Hague WM Higgins J Lowe S McCowan L Oats J Peek MJ Rowan JA Walters B The detection, investigation and management of hypertension in pregnancy: full consensus statement ANZJOG 2000 40 2 139 155 10925900 10.1111/j.1479-828x.2000.tb01137.x

[B35] Kitsantas P Gaffney KF Risk profiles for overweight/obesity among preschoolers Early Hum Dev 2010 86 563 568 10.1016/j.earlhumdev.2010.07.006 20716472

[B36] Rooney BL Mathiason MA Schauberger CW Predictors of obesity in childhood, adolescence, and adulthood in a birth cohort Maternal Child Health J 2010 10.1007/s10995-010-0689-1 20927643

[B37] Rumbold A Crowther CA Hasslam RR Dekker G Robinson JS Vitamins C and E and the risks of preeclampsia and perinatal complications New England Journal of Medicine 2006 354 17 1796 1806 10.1056/NEJMoa054186 16641396

